# Mirizzi Syndrome With a Double Cystic Duct and a Single Gallbladder: A Case Report on a Rare Anatomical Variant and Surgical Challenge

**DOI:** 10.7759/cureus.89216

**Published:** 2025-08-01

**Authors:** Shahzad Yousaf, Mohammad Omar AlShaikh, Amin Abu Hijleh, Ashok Bohra, Ali Reza

**Affiliations:** 1 General Surgery, Mediclinic Parkview Hospital, Dubai, ARE; 2 College of Medicine, Mohammed Bin Rashid University of Medicine and Health Sciences, Dubai, ARE; 3 General and Colorectal Surgery, Mediclinic Parkview Hospital, Dubai, ARE

**Keywords:** anomaly, case report, double cystic duct, gallstones, intraoperative cholangiography, laparoscopic cholecystectomy, mirrizzi syndrome, single gallbladder

## Abstract

While various biliary anomalies have been documented in the literature, their occurrence in clinical practice is uncommon. Common anomalies encountered in practice include variations in cystic duct insertion (such as low or medial insertion), accessory hepatic ducts, and aberrant right hepatic ducts. Less commonly, clinicians may encounter double cystic ducts, duplicated gallbladders, or rare configurations associated with conditions like Mirizzi syndrome. Failure to identify key structures, such as the common hepatic duct (CHD), the common bile duct (CBD), the right hepatic duct (RHD), and the left hepatic duct (LHD), can result in serious intraoperative complications, most notably bile duct injury. Anomalies within Calot’s triangle, in particular, increase this risk during laparoscopic cholecystectomy and underscore the importance of thorough anatomical knowledge and preoperative imaging.

A 45-year-old female presented with symptomatic cholelithiasis and unremarkable laboratory results. She was scheduled for elective laparoscopic cholecystectomy under general anesthesia. During the dissection of Calot’s triangle, an unexpected biliary anomaly was encountered: a double cystic duct in association with Mirizzi syndrome. Dissection was halted, and intraoperative cholangiography (IOC) was performed through the accessory duct. The cholangiogram confirmed a patent and anatomically intact CBD with no evidence of obstruction or injury. Surgery was then completed safely and without complications.

A double cystic duct draining a single gallbladder is an extremely rare anomaly, with approximately only 20 cases described in the literature. These variations are typically not detected preoperatively and are most often discovered during surgery. In such cases, adjunct techniques like IOC or endoscopic retrograde cholangiopancreatography (ERCP) are essential to clarify anatomy and guide safe surgical intervention.

Because preoperative imaging may fail to reveal biliary anomalies, surgeons must maintain a high index of suspicion and proceed with caution when encountering unclear anatomy during cholecystectomy. Selective use of IOC in suspicious cases may help prevent bile duct injury and associated complications, although its routine use remains a topic of ongoing debate in current surgical practice. Vigilance, anatomical awareness, and intraoperative flexibility are key to managing rare biliary variants and ensuring optimal patient outcomes.

## Introduction

Mirizzi syndrome is a rare and challenging complication of gallstone disease, occurring in less than 1% of patients undergoing cholecystectomy. It is caused by an impacted gallstone in the cystic duct or Hartmann’s pouch that compresses the adjacent common hepatic duct (CHD), leading to obstructive jaundice and local inflammation. This chronic inflammatory process can result in dense adhesions, fibrosis, and even cholecystocholedochal fistula formation in advanced cases, as classified by the Csendes system. These changes obscure normal anatomical landmarks, particularly within Calot’s triangle, making dissection hazardous and significantly increasing the risk of bile duct injury [1].

Even more uncommon are anatomical variations, such as a true duplication of the cystic duct, defined as two distinct cystic ducts draining a single gallbladder, an anomaly with approximately 20 cases reported worldwide. This rare biliary variant, characterized by two separate cystic ducts draining a single gallbladder, is often not identified preoperatively and can be easily missed during surgery, further raising the potential for intraoperative complications. Anomalies such as gallbladder or cystic duct duplication can significantly distort normal biliary anatomy, increasing the risk of iatrogenic bile duct injury during cholecystectomy. These variations may go unrecognized preoperatively, especially when asymptomatic, and are often discovered incidentally during surgery. Failure to identify such anomalies can lead to misinterpretation of critical structures, resulting in serious intraoperative complications. Therefore, heightened awareness and careful intraoperative assessment are essential to ensure safe surgical outcomes in the presence of these rare biliary malformations [2].

The coexistence of Mirizzi syndrome with a true duplicated cystic duct is exceedingly rare, with only a handful of reported cases in the literature, and poses significant technical challenges during laparoscopic cholecystectomy. Variants such as duplicated cystic ducts or duplicated gallbladders can distort normal biliary anatomy and complicate dissection within Calot’s triangle, further elevating the potential for bile duct injury [3].

This case underscores the importance of meticulous surgical technique, careful intraoperative assessment, and the sensible use of adjunctive imaging, such as IOC, to safely navigate complex biliary anatomy. It also highlights the value of timely referral to experienced hepatobiliary surgeons or specialized centers when appropriate, to minimize postoperative morbidity.

## Case presentation

A 45-year-old British female, with a known medical history of hypothyroidism and iron deficiency anemia, both of which were well-managed with regular medications, presented to her family physician with complaints of generalized body weakness, loss of appetite, and persistent pruritus (itchiness). She was a non-smoker, did not consume alcohol, and lived with her husband and two children. Initially, the patient visited a family medicine physician in view of her coryzal symptoms and pruritus. Blood investigations revealed mildly elevated liver enzymes, likely due to a viral infection; thus, she was started on symptomatic medical treatment and referred for an abdominal ultrasound to further evaluate the abnormal liver function.

Ultrasonography demonstrated a thin-walled gallbladder with multiple gallstones, the largest measuring up to 7 mm in diameter. No peri-cholecystic fluid or wall thickening was seen, and the liver appeared normal in echotexture and size. Upon further inquiry, the patient reported a long-standing history of indigestion, particularly after fatty meals, as well as occasional episodes of epigastric discomfort. Following the symptomatic treatment regimen, the patient felt better and her liver enzymes normalized; thus, magnetic resonance cholangiopancreatography (MRCP) was not done, as it was not clinically indicated. In view of her symptoms and ultrasound findings, she was referred to a general surgeon, who recommended an elective laparoscopic cholecystectomy with an anticipated one-day hospital admission.

**Figure 1:**
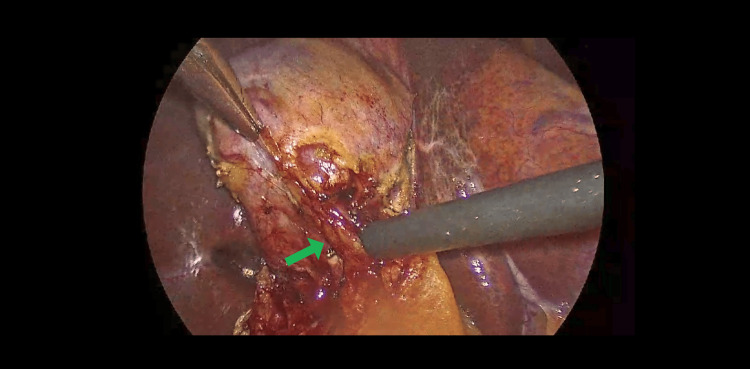
Green arrow points to the cystic artery visualized intraoperatively

**Figure 2:**
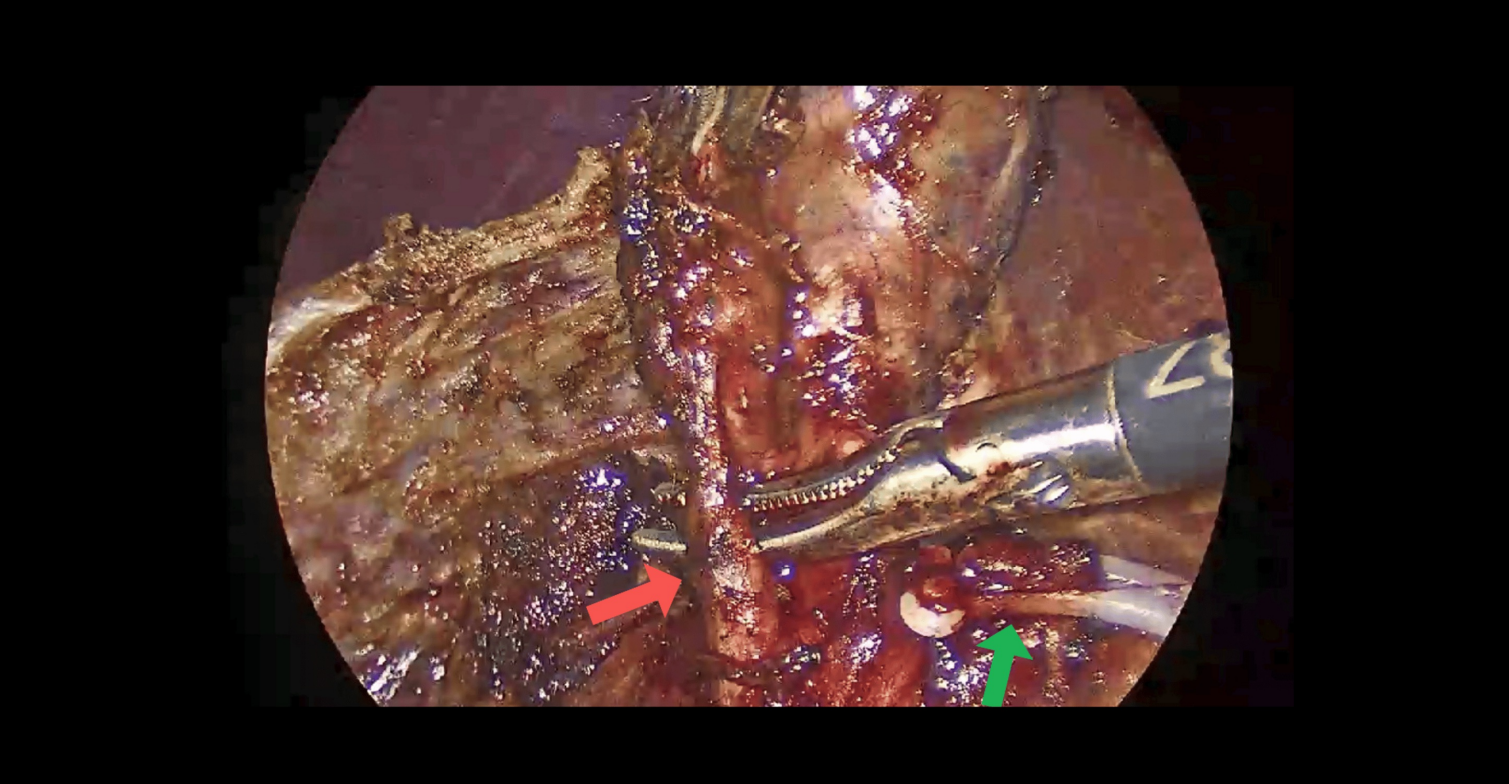
Red arrow points to the first cystic duct visualized intraoperatively. Green arrow points to the clipped and cut end of the cystic artery.

Intraoperatively, dissection of Calot’s triangle proved exceptionally difficult due to unclear anatomical structures. A retrograde (fundus-first) dissection approach was adopted; however, the anatomy remained ambiguous. With meticulous and cautious dissection, the cystic duct and cystic artery were eventually identified and safely ligated using two proximal and one distal Hem-o-lok clip, as seen in Figures 1, 2.

**Figure 3:**
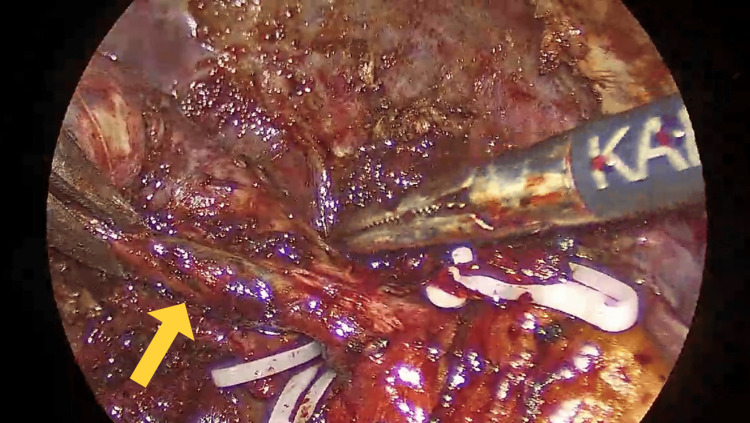
Yellow arrow showing Mirizzi syndrome intraoperatively

**Figure 4:**
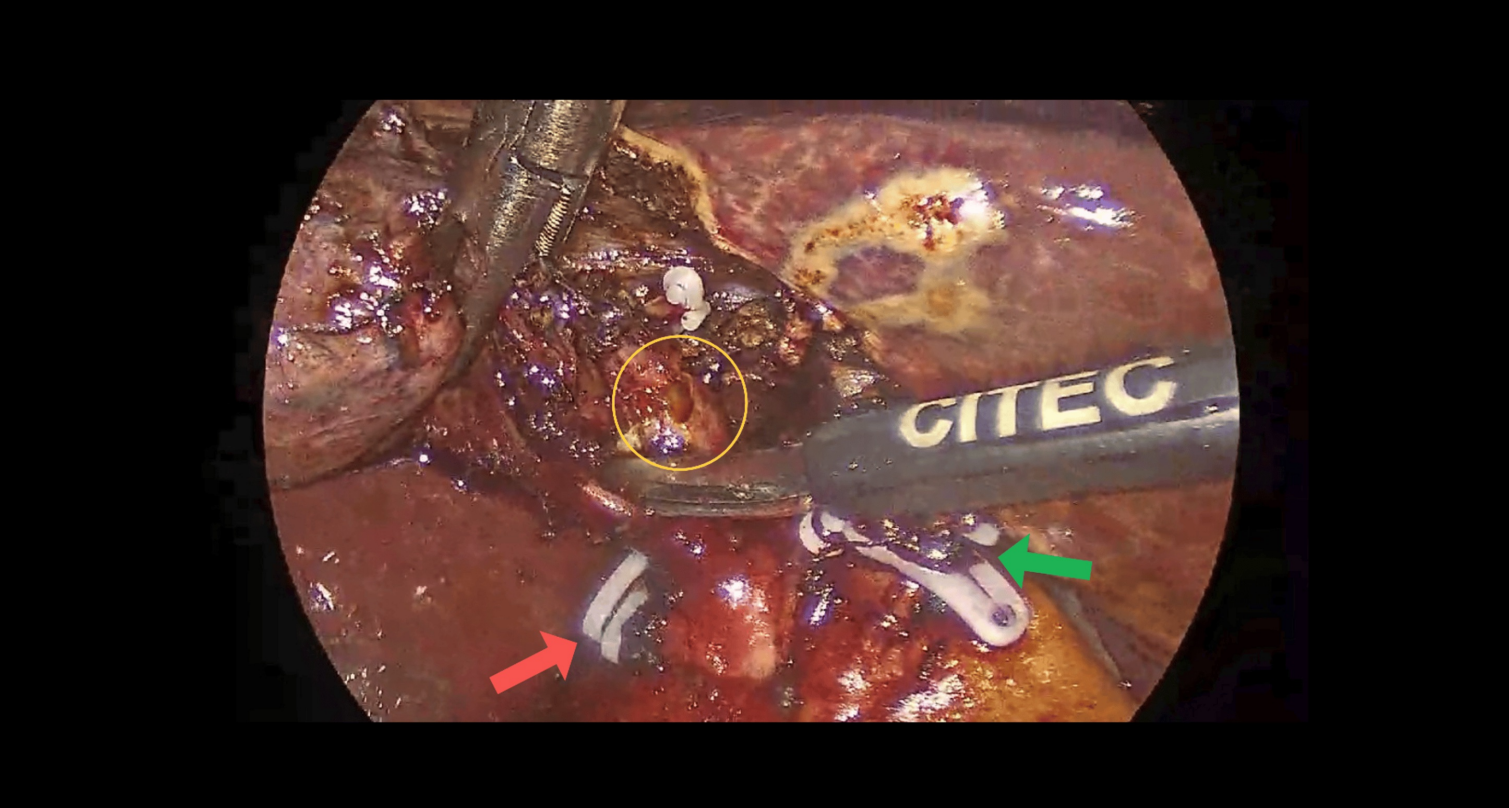
Yellow circle shows the lumen of the second cystic duct identified intraoperatively, red arrow points to the clipped and cut end of the first cystic duct, green arrow points to the clipped and cut end of the cystic artery

**Figure 5:**
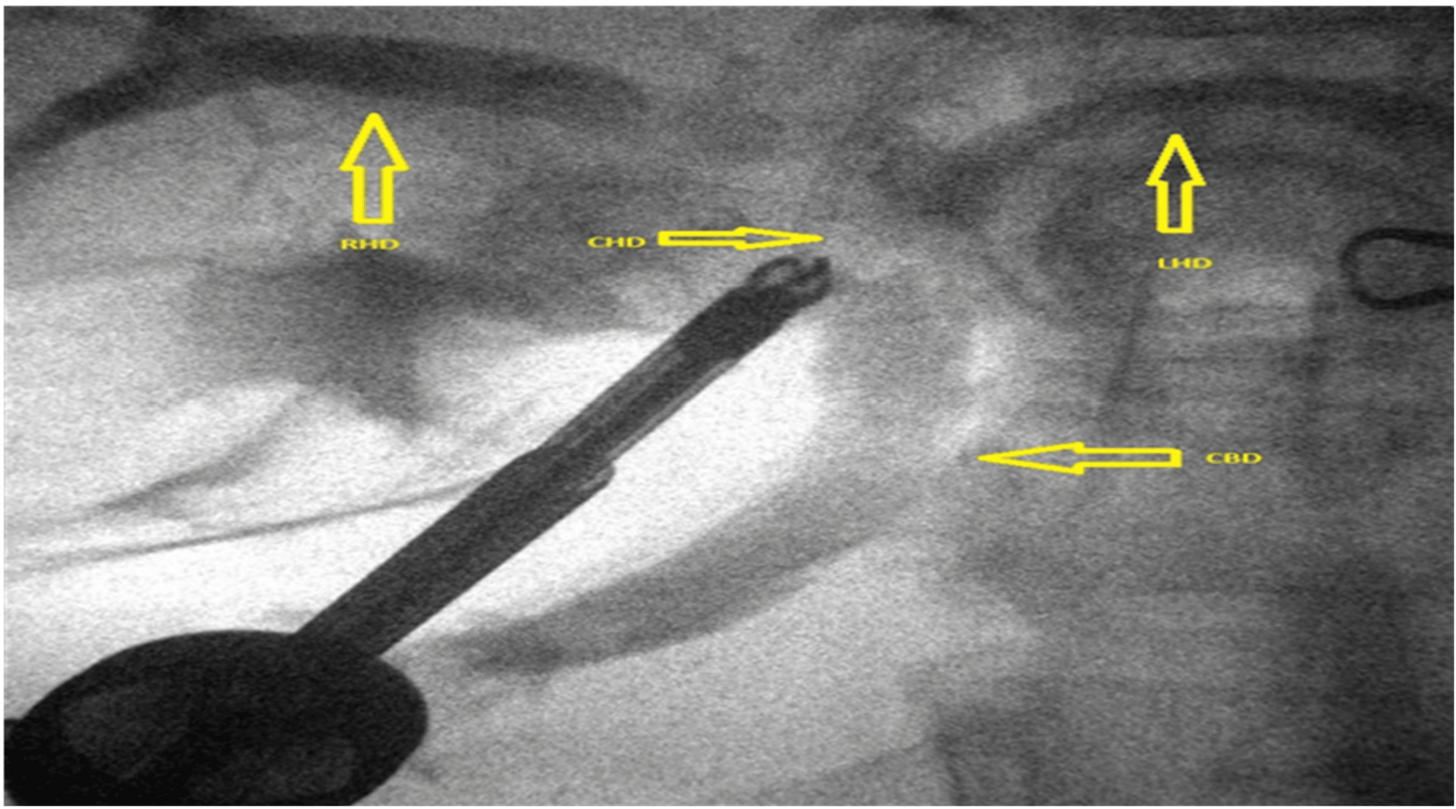
Intraoperative cholangiogram showing RHD (right hepatic duct), LHD (left hepatic duct), CHD (common hepatic duct), and CBD (common bile duct)

Subsequent exploration revealed that Hartmann’s pouch of the gallbladder was closely abutting the CHD, suggestive of Mirizzi syndrome (Figure 3), further complicating the surgery. During the procedure, an additional duct-like lumen draining bile was discovered in continuity with the gallbladder, raising suspicion for an anatomical variant (Figure 4). Due to this unexpected finding, an intraoperative cholangiogram (IOC) was performed, which confirmed the patency and integrity of the common bile duct (CBD), CHD, and both right and left hepatic ducts, as demonstrated in Figure 5.

The lumen was closed securely using Stratafix barbed sutures (Ethicon, Inc., Raritan, NJ, US), and a surgical drain was placed in the operative field for monitoring. The patient had an uneventful postoperative recovery, with stable vital signs and normalizing laboratory parameters. The IOC did not show any biliary leak. In addition, a frozen section was sent, as Mirizzi syndrome elevates the risk of concurrent GB cancer and CBD cancer. The pathology report showed no signs of dysplasia or malignancy. The drain was removed, and she was discharged home after a two-day hospital stay.

At the one-week follow-up, the patient was asymptomatic, her liver function tests were within normal limits, and her overall recovery was considered smooth and uncomplicated.

## Discussion

Anatomical variations within Calot’s triangle are relatively common; however, the presence of a double cystic duct (DCD) originating from a single gallbladder is extremely rare and presents significant challenges during laparoscopic cholecystectomy [4]. A study by Hugh et al. reported that only 33% of individuals display the typical anatomical configuration involving the extrahepatic bile duct, cystic duct, and associated vasculature. The majority (66%) exhibit an angular entry of the cystic duct into the CBD, while 17-20% demonstrate a more parallel alignment, further increasing the risk of misidentification. In 5-8% of individuals, the cystic duct takes a tortuous or spiral course before entering the CBD, and in fewer than 1% of cases, it drains directly into the RHD, further complicating surgical identification and increasing the risk of iatrogenic injury [5].

A DCD is one of the rarest biliary anomalies described, with only a handful of cases reported in the literature [6]. Studies demonstrate a marked female predominance (∼73% of cases) with a highly variable age of presentation, ranging from neonates to elderly patients in their 70s [7-13]. While many cases of DCD are associated with double gallbladders, this is not universal. Al-Otaibi et al. noted that DCD coexists with double gallbladders in up to 80% of cases [14]; however, other reviews found accessory gallbladders in only 33% of cases [15,16].** **In our case, no accessory gallbladder was found, and both cystic ducts originated from a single gallbladder, further underscoring the rarity of this configuration. 

Flannery and Caster classified a DCD into three distinct anatomical types: (1) the H-type, where the accessory cystic duct drains separately into the hepatic duct system (most common); (2) the Y-type, in which both cystic ducts merge into a common channel before entering the biliary tree; and (3) the trabecular type, where the accessory duct drains directly into the liver parenchyma [17]. In our case, the patient’s anatomy correlated with the H type.

The preoperative diagnosis of DCD remains difficult due to its rarity and the limitations of conventional imaging techniques such as ultrasound, CT, or even MRCP. A systematic review found that only 27.3% of DCD cases were diagnosed preoperatively, while a vast majority were detected intraoperatively [3,18]. This highlights the importance of careful surgical dissection and real-time intraoperative evaluation in recognizing unexpected biliary variants.

In our case, the surgery was elective, and no biliary anomalies were detected on preoperative ultrasonography. Intraoperatively, however, we encountered grade II Mirizzi syndrome with inflammation, fibrosis, and compression of the CHD due to an impacted stone in Hartmann’s pouch, which made the dissection particularly complex [3]. Further dissection revealed a second cystic duct, prompting immediate reassessment of the anatomy.

To aid in anatomical clarification and to prevent potential bile duct injury, we performed an IOC. The IOC confirmed the patency of the CBD, CHD, and intrahepatic ducts, and delineated the presence of two cystic ducts draining a single gallbladder. The IOC is widely regarded as a valuable tool for distinguishing ductal anomalies from bile duct injuries during surgery. It not only maps the biliary tree but also detects retained stones and clarifies ambiguous anatomy [19].

Modern intraoperative tools have significantly enhanced the management of complex biliary cases. Indocyanine green (ICG) fluorescence cholangiography provides real-time, non-cannulating visualization of bile ducts, while intraoperative ultrasound (IOUS) proves invaluable when inflammation distorts anatomical planes. Near-infrared (NIR) imaging, often combined with ICG, further improves ductal mapping precision. Both IOC and ICG have demonstrated clear benefits in reducing bile duct injuries and optimizing outcomes for patients with anatomical variants [11]. Although ICG and NIR are not available in our hospital, they can be very useful and beneficial for the doctor during difficult cases, thus lowering patient complications.

## Conclusions

This case highlights the significant surgical challenges encountered during laparoscopic cholecystectomy in the presence of rare biliary anomalies such as Mirizzi syndrome and a true double cystic duct. These anomalies resulted in marked distortion of normal anatomy, necessitating meticulous dissection and the use of IOC to delineate the biliary tree, confirm ductal patency, and prevent iatrogenic injury.

Intraoperative imaging played a critical role in guiding the surgical strategy and avoiding complications such as bile duct injury or postoperative bile leakage. The patient’s favorable postoperative course can be directly attributed to the early recognition of the abnormal anatomy, cautious dissection, and the timely use of IOC. This case underscores the importance of being prepared for unexpected anatomical variations, even during seemingly routine procedures, and supports the selective use of IOC in cases where biliary anatomy is unclear. Surgeons should maintain a high index of suspicion and consider early conversion or referral to a higher-level center when safe identification of structures is compromised.
